# Characterisation and Comparative Evaluation of miRNA Contained in Small Extracellular Vesicles Isolated From Bovine Milk and Whey

**DOI:** 10.1002/jex2.70165

**Published:** 2026-06-30

**Authors:** Caterina Trevisan, Giulia Polacchini, Bruno Stefanon, Monica Colitti

**Affiliations:** ^1^ Department of Agricultural, Food, Environmental and Animal Sciences University of Udine Udine Italy

**Keywords:** bovine, milk, whey, small extracellular vesicles, miRNA

## Abstract

Small extracellular vesicles (EVs) from bovine milk and whey are emerging as biologically active carriers of microRNA (miRNA), yet their composition and variability among different dairy sources remain incompletely understood. We conducted an integrated characterisation of EV‐associated miRNAs isolated from milk and whey collected from three dairy farms. EVs were purified and validated according to MISEV2023 criteria, and small RNA sequencing was performed on milk and whey samples collected on three different days at each farm (18 samples). After quality filtering and adaptor trimming, mapped reads were normalised to counts per million (CPM). To ensure robust detection, only miRNAs present in at least 67% of samples were retained, resulting in a high‐confidence set of 329 miRNAs for downstream analyses. Milk‐derived EVs showed a higher overall miRNA read count than whey‐derived EVs (9.45 ± 5.3 million vs. 5.03 ± 2.5 million reads; *p* < 0.10), with Farm Z displaying the greatest overall abundance. Stability assessment using *Z*‐scored coefficients of variation demonstrated that miRNA reproducibility varied considerably between farms and collections, indicating that farm‐ and collection‐specific factors, rather than matrix origin alone, were major contributors to expression variability. Differential expression analysis (DESeq2) identified 32 miRNAs significantly modulated between milk and whey EVs, with most (*n* = 29) enriched in whey. Functional enrichment of predicted targets indicated involvement in immune and inflammation‐related pathways, including NF‐κB signalling, cytokine–receptor interaction and Ras signalling. Pairwise comparison of farms revealed only a small number of differentially expressed miRNAs in both matrices, and KEGG analyses did not identify significant pathways after multiple‐testing correction. This study presents a comparative overview of bovine milk and whey EV‐associated miRNAs, highlighting higher miRNA abundance in milk, significant farm‐dependent variability, and enrichment of immune‐related pathways that distinguish the two matrices.

## 1 Introduction

Extracellular vesicles (EVs) are membrane‐enclosed nanoparticles released by nearly all cell types and are involved in intercellular communication through the transfer of proteins, lipids and nucleic acids (Raposo and Stoorvogel 2013; Colombo et al. 2014). According to the Minimal Information for Studies of Extracellular Vesicles (MISEV2023) guidelines, EVs consist of heterogeneous subpopulations, including small EVs (typically 30–150 nm in diameter; mean size ∼ 100 nm), which are commonly enriched in 100,000 × *g* ultracentrifugation pellets (Welsh et al. 2024). These vesicles carry diverse RNA species, including microRNAs (miRNAs), which can modulate gene expression in recipient cells and influence immune, developmental and metabolic pathways.

Among biological fluids, milk is one of the richest and most accessible natural sources of EVs. Bovine milk is produced in large volumes worldwide, making it particularly attractive for nutritional, biomedical and industrial applications (Chen et al. 2010). Milk‐derived EVs have been shown to withstand gastrointestinal conditions and deliver bioactive cargo to intestinal and systemic tissues, exerting biological effects following oral administration (Liao et al. 2017; Benmoussa et al. 2019). These properties support their proposed roles in immune modulation, gut barrier regulation and metabolic homeostasis. In addition to their physiological relevance, milk‐derived EVs are increasingly explored as natural nanocarriers for therapeutic molecules and RNA‐based interventions due to their stability, low immunogenicity and scalability (Kong et al. 2025). Moreover, growing evidence supports their immunomodulatory and protective effects in preclinical models (Di et al. 2024). A central component of EV biological activity is their miRNA cargo. Milk EV‐associated miRNAs are evolutionarily conserved across mammals and include abundant families such as let‐7, miR‐148, miR‐26 and miR‐200, which are implicated in epithelial homeostasis, immune regulation and developmental signalling (van Herwijnen et al. 2018; Stefanon et al. 2023).

However, despite growing interest, several aspects of milk EV‐miRNA biology remain unresolved. Methodological variability in EV isolation workflows, differences in milk processing, and incomplete bovine miRNA annotation complicate direct comparisons between studies. Furthermore, the extent to which EV‐miRNA composition varies across production contexts or milk fractions remains insufficiently characterised (Ansari et al. 2024).

Whole milk is a complex biological matrix containing fat globules, casein micelles, whey proteins, and cellular debris, all of which may influence EV recovery and purity. During cheese‐making, milk is enzymatically coagulated to separate curd from liquid whey. Whey is therefore a derivative fraction of milk that contains soluble proteins, lactose and residual vesicles but lacks most fat globules and casein micelles. These compositional differences may affect both EV isolation efficiency and vesicle‐associated cargo. Whey has been proposed as a more tractable matrix for large‐scale EV recovery due to its lower lipid and casein content, which reduces interference during ultracentrifugation and filtration workflows (Kleinjan et al. 2021). In line with this, del Saz‐Lara et al. (2025) recently identified milk and cheese‐making whey as sustainable and scalable sources of extracellular vesicles, demonstrating the feasibility of large‐scale isolation methods with potential industrial and therapeutic applications. However, industrial processing steps such as heating, homogenisation and storage have been shown to alter EV integrity, molecular marker profiles, and RNA stability in bovine milk, potentially affecting downstream isolation efficiency and cargo interpretation (Kleinjan et al. 2021; Colella et al. 2024). Various isolation workflows have been developed for milk‐derived EVs, including differential ultracentrifugation, precipitation‐based methods, tangential flow filtration and electrophoretic‐assisted membrane technologies (Vaswani et al. 2017, Vaswani et al. 2019; Ko et al. 2023). Nevertheless, methodological heterogeneity remains a major source of variability across studies, complicating direct comparisons of EV yield and molecular cargo. Despite the expanding literature on milk‐derived EVs, direct comparative analyses of EV‐associated miRNA profiles between whole milk and the corresponding whey fraction remain limited and incompletely characterised. In particular, it is unclear whether differences between these matrices are primarily due to compositional factors intrinsic to the milk fraction or to external variables such as farm management, herd characteristics or collection timing. Clarifying this distinction is essential for both biological interpretation and translational applications, including biomarker discovery and industrial EV recovery strategies.

In this context, the present study aimed to conduct an integrated comparative characterisation of EV‐associated miRNAs isolated from bovine whole milk and the corresponding whey obtained during cheese‐making processes at three dairy farms. By combining standardised EV isolation procedures, physicochemical characterisation in accordance with MISEV2023 recommendations, and small RNA sequencing, we aimed to (i) identify shared and matrix‐specific miRNA signatures between milk and whey EV preparations and (ii) assess the extent of farm‐dependent variability relative to matrix‐dependent differences. This approach provides insight into the consistency and biological relevance of bovine milk‐ and whey‐derived EV miRNA cargo and contributes to establishing a comparative framework for future functional and translational investigations.

## 2 Materials and Methods

### 2.1 Sample Collection

Bulk milk was collected after the morning milking from the tanks of three dairy farms in the Friuli Venezia‐Giulia region (Italy), named Z, S and L, which rear Simmental cows (*Bos taurus*). Milk and whey samples were collected as part of routine dairy farm production, and no additional procedures were performed on animals specifically for this study. Therefore, no experimental animal ethics approval was required.

The collected milk was processed on each farm for cheese production using standard enzymatic coagulation procedures. Whey samples were taken from the corresponding milk batches used for cheese‐making on the same day as milk sampling. During cheese‐making, milk undergoes enzymatic coagulation (rennet‐induced), which separates the solid curd (casein‐rich fraction) from the liquid whey. Whey thus represents the soluble fraction of milk, containing lactose, whey proteins and residual vesicles, but lacking most fat globules and casein micelles. Whey samples were collected immediately after curd separation and before any further processing steps (such as heating, salting or fermentation), ensuring comparable collection conditions across farms.

A total of 1000 mL of bulk milk or whey was collected per sample. Each farm provided three samples of milk and three samples of whey, collected on three different days within 1 week, for a total of 18 samples, each representing three independent batches of both matrices, as shown in Table 1.

**TABLE 1:** List of milk and whey samples collected from the three farms.

Sample ID	Source	Farm	Collection
L1Z	milk	Z	1
L2Z	milk	Z	2
L3Z	milk	Z	3
L1L	milk	L	1
L2L	milk	L	2
L3L	milk	L	3
L1S	milk	S	1
L2S	milk	S	2
L3S	milk	S	3
S1Z	whey	Z	1
S2Z	whey	Z	2
S3Z	whey	Z	3
S1L	whey	L	1
S2L	whey	L	2
S3L	whey	L	3
S1S	whey	S	1
S2S	whey	S	2
S3S	whey	S	3

The number of lactating animals contributing to bulk milk production was 25 (Farm Z), 69 (Farm S) and 59 (Farm L), as detailed in Table S1, which also summarises herd composition, including the proportion of primiparous and multiparous cows, dry animals, and the average age and milk yield of primiparous and multiparous cows for each farm. Bulk milk composition is provided in Table S2. Samples were frozen at −20°C within 24 h of collection.

### 2.2 EV Isolation

Isolation protocols include an initial sample cleaning step to remove fat globules and cellular debris. Bulk milk or whey (1000 mL per sample) was subjected to sequential clarification steps: centrifugation at 5000 × *g* for 30 min at 4°C, followed by collection of the supernatant and a second centrifugation at 12,000 × *g* for 1 h at 4°C to remove residual fat globules and cellular debris. After clarification, approximately 200 mL of defatted milk or whey per sample was processed for EV isolation and stored at −80°C until further processing. The remaining clarified material was divided into aliquots (approximately 200 mL each) and stored at −80°C without further processing. All samples were subjected to identical freeze–thaw conditions prior to ultracentrifugation. Samples derived from whole milk and processed as described below are hereafter referred to as milk‐EVs, whereas samples derived from whey and processed using the same ultracentrifugation workflow (without isoelectric precipitation) are referred to as whey‐EVs.

Before ultracentrifugation, defatted milk samples were thawed and subjected to isoelectric precipitation to remove residual caseins. Specifically, samples were diluted 1:1 with distilled water, incubated at 37°C for 10 min, and adjusted to pH 4.6 with 6 N HCl. The mixture was centrifuged at 10,000 × *g* for 20 min, and the supernatant was collected (Cintio et al. 2020; Stefanon et al. 2023). Whey samples were not subjected to isoelectric precipitation.

The resulting supernatant was stored at –80°C before the final ultracentrifugation. Although freeze–thaw cycles may affect EV integrity and recovery, as discussed in recent systematic reviews (Ahmadian et al. 2024), all samples were subjected to identical storage conditions, minimising systematic bias between matrices.

Ultracentrifugation was performed using a Beckman Optima LE‐80K ultracentrifuge (Beckman Coulter S.r.l., Milan, Italy) equipped with a Beckman TYPE 70 Ti rotor (k‐factor: 44). Samples were loaded into polycarbonate tubes with a maximum capacity of 26.3 mL (Beckman Coulter). Clarified supernatants were divided into multiple tubes to accommodate the processed volume (200 mL per sample). Final ultracentrifugation was carried out at 100,000 × *g* for 1 h at 4°C. Pellets were resuspended in 150 µL phosphate‐buffered saline (PBS 1×) or 300 µL RIPA lysis buffer (1×), depending on downstream applications.

### 2.3 Transmission Electron Microscopy (TEM)

EV preparations were quantified by protein assay and diluted in distilled water to obtain a final amount of 200 ng in 500 µL per sample before grid preparation. Samples were processed by the Dibio Imaging Facility at the Department of Biology, University of Padua (Italy) for TEM acquisition. Vesicle preparations (10 µL) were adsorbed onto glow‐discharged (Leica EM ACE 600) 400‐mesh carbon‐coated copper grids, then negatively stained with 2% aqueous uranyl acetate. Samples were subsequently examined with a Tecnai G2 (FEI) transmission electron microscope operating at 120 kV, and digital images were acquired using a Veleta digital camera (Olympus Soft Imaging Solutions).

### 2.4 Nanoparticle Tracking Analysis (NTA)

Particle size distribution and concentration were determined using a Nanosight LM10 (Malvern System Ltd., London, UK), equipped with a 405‐nm laser and sCMOS camera. Samples were diluted 1:100,000 in PBS prior to analysis to achieve measurable particle concentrations within the optimal detection range recommended by the manufacturer. Measurements were performed at 22°C with viscosity set to 1.0 cP in the analysis software, approximating the viscosity of PBS at room temperature. Videos were captured at 25 frames per second with a camera level of 14, slider shutter 1259 and slider gain 366. For each sample, three 60‐s videos (1499 frames) were recorded under identical acquisition settings. Data were analysed using NTA software version 3.2 Dev Build 3.2.16 with a detection threshold of 5 and automatic blur size and maximum jump distance settings. Particle concentration and size distribution parameters (mean, mode, D10, D50 and D90) were calculated from merged data. Particle counts during acquisition ranged from approximately 28 to 173 particles per frame across all milk and whey samples, remaining within the operational measurement range recommended for NTA analysis.

### 2.5 Protein Quantification and Western Blotting

Total proteins were extracted in RIPA 1× lysis buffer with protease and phosphatase inhibitors and quantified by the Bradford assay using a bovine serum albumin standard. Absorbance was measured at 595 nm using the Spark microplate reader (Tecan Group Ltd., Switzerland). Control samples corresponded to the post‐ultracentrifugation supernatant (UC supernatant) fraction. These samples were processed in parallel with EV pellets and subjected to the same protein extraction protocol using RIPA buffer with inhibitors before quantification.

Total proteins (20 µg per lane) were separated by SDS–PAGE using handmade polyacrylamide gels prepared with a 37.5:1 acrylamide:bis‐acrylamide solution (Hoefer). Resolving gels (10%–13%, as indicated for each target protein) were cast using a SE250 Mighty Small II Mini Vertical Protein Electrophoresis Unit (Hoefer, USA). Electrophoresis was performed in Tris–glycine–SDS running buffer under constant voltage, initially at 150 V and then increased to 200 V. The system was powered by a PowerPac HC High‐Current Power Supply (Bio‐Rad, USA). A chiller unit set at 15°C recirculated cooling water to prevent gel overheating during electrophoresis. After transfer onto PVDF membranes (Immobilon‐P; Millipore, Darmstadt, Germany) using the Biometra Fastblot semi‐dry apparatus (Analytik Jena GmbH+Co, Jena, Germany), samples were probed for markers commonly enriched in small EVs with the following antibodies: Mouse anti‐CD63 (ab193349, Abcam, Cambridge, MA, USA), Rabbit anti‐CD81 (bs‐6934R, Bioss) and Rat anti‐HSC70 (ab19136, Abcam, Cambridge, MA, USA). Negative controls included Rabbit anti‐GM130 (bsm‐61120R, Bioss) and Goat anti‐lactoferrin (A10‐126A, Bethyl). Secondary antibodies were HRP‐conjugated. Detection was performed using ImageQuant LAS 500 (GE Healthcare Life Sciences, Hatfield, UK).

### 2.6 RNA Extraction

RNA was extracted using the mirVana miRNA Isolation Kit (Thermo Fisher, Milan, Italy). The lysate was mixed with 30 µL of homogenate additive, incubated on ice for 10 min, and extracted once with 300 µL of acid phenol:chloroform. Samples were centrifuged, and the aqueous phase was collected in a new tube. After washing with 100% absolute ethanol at room temperature, the lysate–ethanol mixture was placed in a filter cartridge and centrifuged until all the lysate solution had passed through the filter. The filter cartridge was then washed repeatedly before 40 µL of RNase‐free water preheated to 95°C was added. miRNAs were collected by centrifugation for 25 s at 10,000 *g*. RNA concentration and purity were evaluated using a Spark microplate reader (Tecan Group Ltd., Switzerland).

### 2.7 Small RNA Sequencing and Bioinformatic Processing

RNA purity, integrity and concentration were assessed using an Agilent 2100 Bioanalyzer (Agilent Technologies). Only samples meeting strict quality thresholds (RNA Integrity Number, RIN > 7; rRNA 28S/18S ratio > 1.8) were retained for library construction. Small RNA libraries were prepared using the Small RNA Library Prep Kit for Illumina (Abclonal, RK20305) following standard procedures: 3′ and 5′ adapters were ligated to small RNA ends, first‐strand cDNA was synthesised via reverse transcription primer hybridisation, and double‐stranded cDNA libraries were generated through PCR amplification. Libraries were pooled and sequenced on an Illumina NovaSeq 6000 platform (Illumina Inc., USA) using single‐end 50 bp reads (SE50). Raw FASTQ files were processed by Novogene using fastp for quality filtering and adapter trimming. Reads containing adapter contamination, ambiguous bases (*N* > 10%), or low‐quality bases (Phred score < 20) were removed prior to downstream analyses.

Clean reads were mapped to the *Bos taurus* reference genome (Ensembl release 109, ARS‐UCD1.2) using Bowtie (v0.12.9) with the parameters ‐v 0 ‐k 1. Known and novel miRNAs were identified using miREvo (v1.1) integrated with miRDeep2 (v0.0.5) and ViennaRNA (v2.1.1). Differential expression analysis between biological groups was performed using DESeq2 (v1.12.0) with adjusted *p* values (*p*adj < 0.05).

Raw sequencing data have been deposited in the NCBI Sequence Read Archive (BioProject ID: PRJNA1381537).

### 2.8 Read Quality Control and Small RNA Annotation

Sequencing of the 18 exosomal RNA libraries generated 6–16 million raw reads per sample, consistent with typical yields for small RNA sequencing. Artefactual sequences, including 5′ primer contaminants, adaptor dimers, no‐insert tags, poly(A)‐rich reads, oversized inserts and short non‐informative tags, were removed through sequential filtering steps.

The proportion of low‐quality or ambiguous reads in the samples was <0.1%, indicating excellent sequencing performance. After filtering, the percentage of clean reads suitable for annotation ranged from ∼67% (L3S) to ∼97% (L2Z), with the majority exceeding 90%, confirming the technical robustness of the workflow.

Clean reads were used for small RNA annotation, yielding 1031 unique small RNA (sRNA) sequences, including canonical microRNAs (miRNAs), tRNAs, snRNAs, snoRNAs, rRNA fragments, repeat‐associated RNAs, exonic and intronic fragments and novel miRNAs.

### 2.9 MiRNA Filtering and Abundance Quantification

Mapped miRNA read counts were converted to counts per million (CPM) for descriptive analyses. To ensure robust representation across the dataset, only miRNAs detected in at least 67% of samples were retained, resulting in a high‐confidence set of 329 miRNAs. This prevalence filter is widely used in sRNA‐seq pipelines to remove sporadically detected or low‐abundance miRNAs and to improve comparability across biological groups (Baran‐Gale et al. 2015; Srinivasan et al. 2019). CPM was calculated after filtering, and these values were used exclusively for abundance profiling and stability analyses, not for differential expression.

### 2.10 Stability *Z*‐Score Analysis

To assess the reproducibility of miRNA expression between farms and collections, a stability analysis based on *Z*‐scored coefficients of variation (CV) was conducted. For each matrix × farm × collection group, mean CPM values were calculated for the set of priority miRNAs. The CV was used as an index of intra‐group variability, and values were standardised relative to the global mean CV to generate stability *Z*‐scores. Negative *Z*‐scores indicate greater expression stability, while positive values reflect higher variability.

### 2.11 Differential Expression Analysis

Differential expression (DE) was assessed using DESeq2 applied to raw read counts, in accordance with the negative binomial modelling required by the method (Love et al. 2014). miRNAs with an adjusted *p* value < 0.05 (Benjamini–Hochberg correction) and an absolute log_2_ fold change ≥ 1 were considered significantly differentially expressed.

After prevalence filtering (≥67% of samples), 329 miRNAs were retained for differential expression analysis. In the milk versus whey comparison, 32 miRNAs met the predefined statistical criteria for differential expression (adjusted *p* < 0.05; |log_2_FC| ≥ 1), including 3 with higher expression in milk and 29 with higher expression in whey.DE analyses were performed for milk versus whey EVs and for all pairwise farm comparisons (L, S, Z) within each matrix. Only *Bos taurus*–annotated miRNAs were used for downstream functional enrichment; novel or non‐annotated miRNAs were retained for DE testing but excluded from pathway analysis.

### 2.12 Functional Enrichment Analysis

Functional interpretation of DE miRNAs was performed using miRNet 2.0 (https://www.mirnet.ca) (Chang et al. 2020). As pathway analysis requires species‐specific miRNA–target interactions, only *Bos taurus* miRNAs with available target predictions were included. Target genes were obtained from the miRanda database, which provides the most comprehensive bovine coverage; experimentally validated resources (TarBase, miRTarBase) were excluded due to limited annotation for cattle.

KEGG pathway enrichment (Kyoto Encyclopedia of Genes and Genomes) was conducted using cumulative hypergeometric testing. *p* values were adjusted using the False Discovery Rate (FDR, Benjamini–Hochberg method); values less than 0.05 were considered significantly enriched. Volcano plots and summary tables were generated in R.

To provide miRNA‐level functional annotation, Gene Ontology (GO) Biological Process enrichment analysis was additionally performed on miRanda‐predicted target genes using the STRING database (v11.5) implemented in Cytoscape (stringApp). Multiple testing correction was applied using the FDR, and GO terms with FDR < 0.05 were considered significant. Only representative non‐redundant GO Biological Process terms were retained for reporting.

### 2.13 Statistical Analysis

Modal particle size, particle concentration and protein concentration of EXO from milk and whey were compared using an unpaired Student's *t*‐test. Comparisons of total filtered miRNA reads between milk and whey‐EVs were assessed using both Welch's *t*‐test and the non‐parametric Mann–Whitney U test. Farm‐specific comparisons (milk vs. whey within each farm and pairwise farm comparisons within each matrix) were evaluated using Mann–Whitney U tests due to the small sample size (*n* = 3 per group) and non‐normal data distributions. Statistical significance was defined as *p* ≤ 0.05, while values between 0.05 and 0.10 were interpreted as trends. All descriptive statistical analyses were performed using GraphPad Prism v9.0 (GraphPad Software Inc., La Jolla, USA).

## 3 Results

### 3.1 Nanosight Analysis and TEM Analyses

**FIGURE 1:**
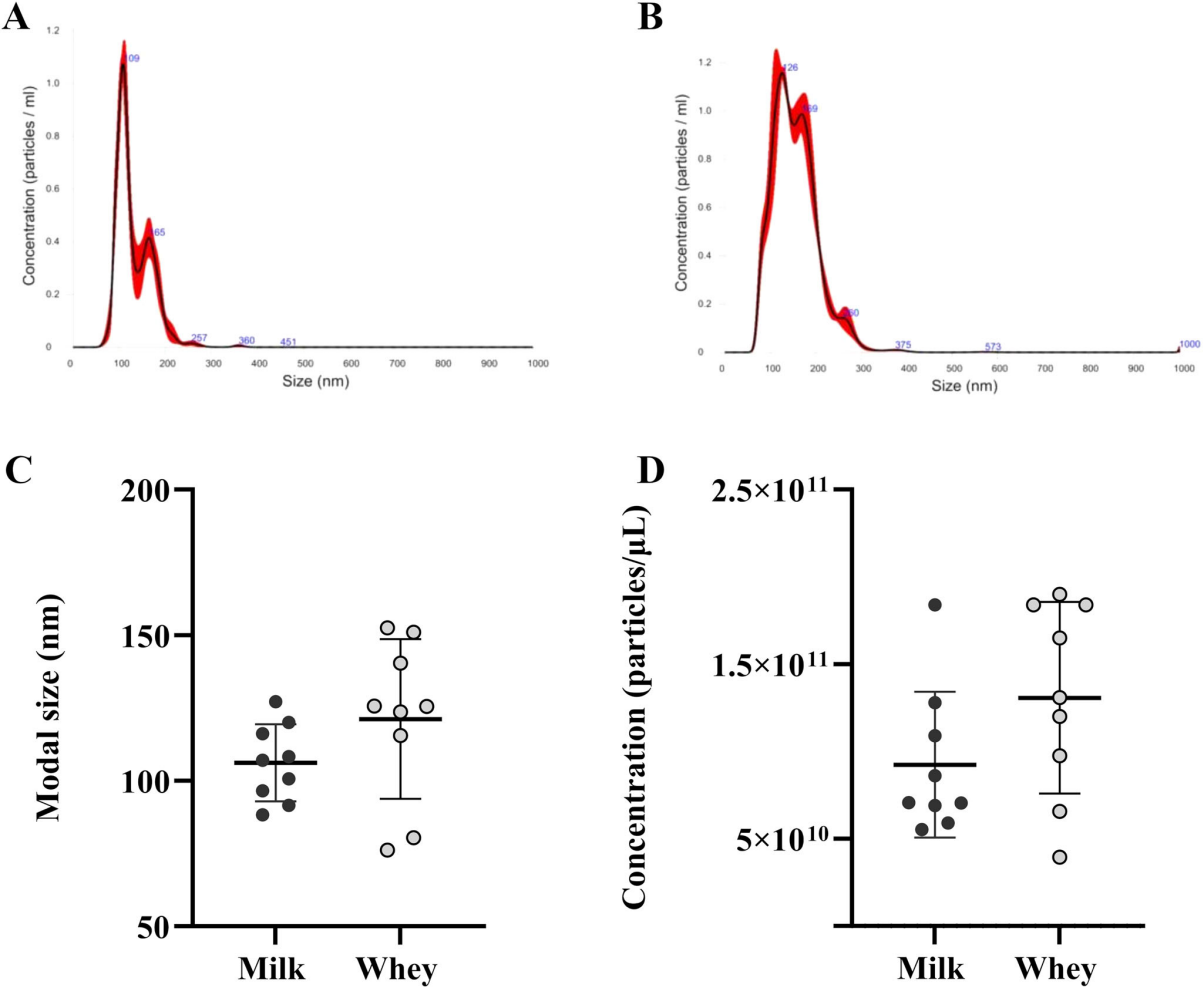
Nanoparticle tracking analysis (NTA) of EVs isolated from bovine milk (milk‐EVs, A) and whey (whey‐EVs, B). The size distribution profiles show a predominant population of vesicles with diameters between 30 and 150 nm, consistent with small EV dimensions. The y‐axis represents particle concentration (particles/mL × 10^7^). Concentrations were corrected for the dilution factor (1:100,000). The modal size (C) and concentration (D) of EVs isolated from bovine milk and whey as determined by nanoparticle tracking analysis. Data are expressed as mean ± SD from *n* = 9 per group. Statistical analysis was performed using an unpaired Student *t*‐test.

The modal size and concentration of the biological particles in the obtained vesicular precipitates were measured using the Nanosight system. Figure 1 shows representative images of the frequency distributions of particles derived from milk (Figure 1A) and whey (Figure 1B). Vesicles isolated from both matrices had modal diameters between 88 and 153 nm, consistent with the exosome range (30–150 nm). Concentrations ranged from 10^10^ to 10^1^ ^1^ particles/µL, with no significant variations among farms or replicates.

In addition to the modal size values, the size distribution profiles revealed distinct subpopulations within both milk and whey EV preparations. In both matrices, prominent peaks were observed between approximately 110–125 nm and around 165 nm, suggesting the presence of heterogeneous small EV subsets. Notably, whey‐derived EV samples showed a relatively higher intensity of larger particles around ∼250–270 nm compared with milk‐derived EVs. These differences were not fully captured by modal diameter values alone (Figure 1C), highlighting the importance of analysing the complete size distribution curves. The presence of larger particles in whey may reflect matrix‐specific differences in vesicle composition or residual co‐isolated particles.

Overall, evaluating the obtained data without distinguishing between different farms, milk‐derived vesicles exhibited a slightly smaller diameter than whey‐derived vesicles (Figure 1C), while the concentration was comparable between particles from both matrices (Figure 1D).

**FIGURE 2:**
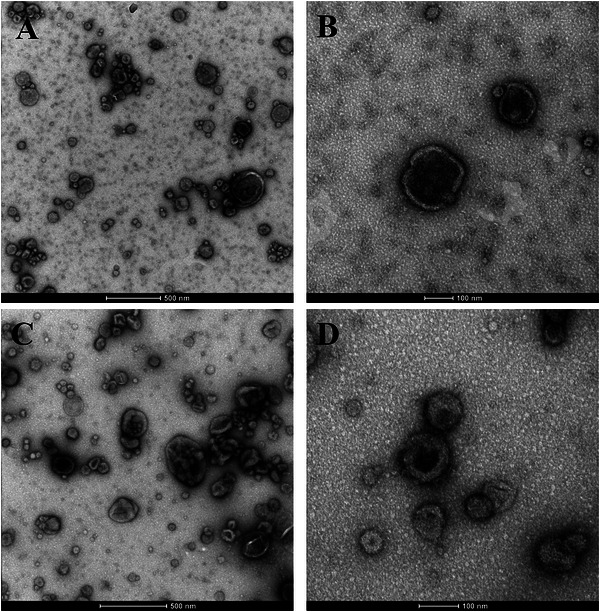
Representative transmission electron microscopy (TEM) images of EVs isolated from bovine milk (A, B) and whey (C, D). Multiple imaging areas were examined for each matrix to assess vesicle morphology and distribution. The vesicles display round to slightly ovoid morphology within the size range typical of small EVs. Scale bars: 500 nm (A, C); 100 nm (B, D).

Figure 2 presents representative images of the characterisation performed using TEM analysis on samples derived from milk (Figure 2A, B) and whey (Figure 2C, D **)**. The observed vesicle population is consistent with the expected morphological features of small EVs. Multiple TEM fields were examined for each matrix to assess vesicle morphology and distribution. Widefield images (500 nm scale) confirmed abundant EV‐like particles with minimal debris, while higher magnification images (100 nm scale) revealed vesicles with round to slightly ovoid morphology and defined membrane boundaries. Only occasional larger vesicles were observed, and overall, the preparations displayed limited proteinaceous or membranous contamination, supporting the high purity of the isolated EV fraction. The observed size heterogeneity was consistent with the NTA size distribution profiles.

### 3.2 Protein Yield and Purity

The total protein content of all vesicular precipitates obtained from milk and whey was quantified, ranging from 2.13 to 9.45 µg/µL. The whey‐derived vesicles had a significantly higher protein concentration (*p* < 0.05), averaging 6.17 µg/µL, compared to the milk‐derived vesicles, which had an average protein content of 3.71 µg/µL. To complement the protein quantification and provide an additional indicator of preparation purity, the particle‐to‐protein ratio was calculated as (particles/mL)/(µg protein/mL), using NTA‐derived particle concentrations corrected for the 1:100,000 dilution factor. Milk‐EVs exhibited an average ratio of approximately 2.49 × 10^10^ particles per microgram of protein, whereas whey‐EVs showed a comparable ratio of approximately 2.40 × 10^10^ particles per microgram of protein. Although NTA quantifies total particles rather than EV marker‐positive vesicles, these values suggest a similar particle‐to‐protein relationship between the two matrices.

**FIGURE 3:**
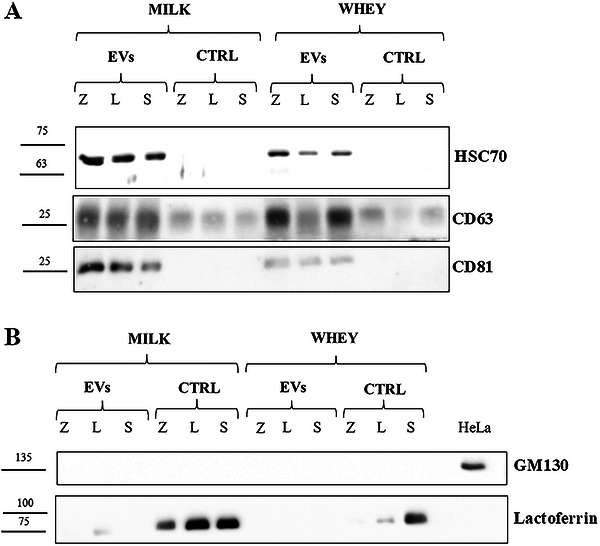
Representative image of Western blot analysis of milk‐ and whey‐derived EVs. (A) EV preparations from the three farms (Z, L, S) showed enrichment of markers commonly found in small EVs, HSC70, CD63 and CD81, which were absent in the corresponding CTRL fractions. (B) The Golgi marker GM130 was not detected in any EV or CTRL sample; HeLa lysate served as a positive control. Lactoferrin was abundant in milk CTRL samples but showed limited detection in EV preparations under the applied ultracentrifugation conditions.

Subsequently, 20 µg of protein extract from each of the 18 EV preparations (9 milk and 9 whey) was analysed by Western blot to assess the expression of vesicular and non‐vesicular markers, and a representative image is shown in **Figure** **3**. Marker expression was evaluated in the EV preparations and their respective negative controls (CTRL), corresponding to the final supernatant fraction collected after ultracentrifugation. This analysis showed that samples derived from milk and whey from the different farms (Z, L, S) were consistently enriched in vesicles, as demonstrated by the presence of markers commonly enriched in small EVs, including HSC70, CD63 and CD81 in all EV preparations and their absence in the corresponding CTRL samples.

To further assess sample purity, the Golgi marker GM130 was examined, as its absence indicates minimal contamination by cellular components. GM130 was not detected in either EV or CTRL samples. Therefore, a HeLa cell lysate was included as a positive control to confirm antibody specificity. The lack of a GM130 signal in all vesicle preparations supports the absence of Golgi‐derived contaminants.

Lactoferrin (LTF), a highly abundant whey protein with antimicrobial and immunomodulatory properties, was strongly detected in the CTRL fractions but only faintly in EV pellets. The association of LTF with milk‐derived EVs remains debated in the literature. Some studies report partial co‐isolation of abundant milk proteins with EV fractions depending on the isolation strategy (Vaswani et al. 2017; Sedykh et al. 2019), while others suggest that most LTF remains soluble and is not stably incorporated into EV membranes (van Herwijnen et al. 2018). Processing conditions, including heat treatment and matrix handling, have been shown to influence EV composition and co‐isolated protein profiles (Kleinjan et al. 2021). In the present study, the presence of both CD63 and LTF in the post‐ultracentrifugation supernatant indicates that LTF was not efficiently co‐pelleted with CD63‐positive EVs under the applied isolation conditions (100,000 × *g*, 1 h). Extended ultracentrifugation times could potentially improve the recovery of smaller EV populations (<100 nm diameter) and increase the detection of LTF within EV isolates. However, based on the current experimental design, it is not possible to conclusively determine whether LTF is genuinely associated with EVs or represents a co‐isolated non‐vesicular component.

These results support the successful enrichment of EVs from both milk and whey matrices, regardless of the farm (Z, L or S) or the specific bulk milk and whey sampling.

### 3.3 Distribution and Variability of Small RNA Categories

Among all categories, known miRNAs constituted the most abundant fraction in both milk and whey EVs, although their proportions varied considerably between samples. In milk EVs, the proportion of known miRNAs ranged from 29.7% to 75.8%, whereas in whey EVs it ranged from 15.8% to 66.8%. In contrast, rRNA, tRNA, snRNA and snoRNA together accounted for only a minor portion of the libraries, each representing less than 5% of total reads.

In addition to canonical miRNAs, a substantial proportion of clean reads mapped to other RNA classes. On average, exonic sequences accounted for approximately 4%–5%, while intronic fragments represented about 12%–14%, although some samples showed higher values due to biological or technical variability. Repeat‐associated sequences contributed a further ∼4%–5% of the total read count. The ‘other’ category, which includes unannotated or poorly characterised small RNAs and RNA fragments not assigned to defined sRNA classes, constituted the largest non‐miRNA component of the dataset. This category ranged from 20% to 55% of total reads, with a mean of ∼38%–40%, consistent with the molecular heterogeneity typically observed in extracellular vesicle‐derived sRNA profiles. Together, these results indicate that EV RNA cargo comprises a complex mixture of small RNA species beyond miRNAs, reflecting the biological diversity characteristic of EV‐associated RNA.

The proportion of known miRNAs in milk‐derived EVs varied considerably among samples, ranging from approximately 30% (L2L) to about 75% (L3Z), with L1L also showing a relatively high proportion (around 57%). In whey‐derived EVs, known miRNAs were generally less abundant, most often between 15% and 40%, with the notable exception of S1Z, which reached approximately 67%. This pattern suggests that whey‐EVs have a more heterogeneous and transcriptionally noisy small‐RNA profile compared with milk‐EVs.

The fraction of rRNA‐derived reads remained consistently low (typically less than 2%–3%) in both matrices, supporting the high purity of the RNA preparations. However, certain samples, most notably L2L (approximately 22%) and S3L (around 21%), showed increased representation of intronic and exonic fragments, indicating the presence of residual genomic RNA fragments or background RNA species not belonging to canonical small‐RNA classes.

The proportion of reads classified as ‘other’, representing unannotated or poorly characterised transcripts, also varied widely among samples, ranging from ∼20% in L3Z to nearly 50% in S3S, consistent with the molecular heterogeneity expected in EV‐derived RNA.

Overall, L3Z and L1L showed the highest enrichment of canonical miRNAs, suggesting that these milk‐derived EV fractions may contain a more specific and biologically interpretable small RNA cargo compared with whey‐derived EVs.

### 3.4 MiRNA Abundance in Milk‐EVs and Whey‐EVs

Milk‐EVs contained an average of 9.45 ± 5.3 million mapped miRNA reads (mean ± SD; *n* = 9), while whey‐EVs contained 5.03 ± 2.5 million reads (*n* = 9). Although the difference was not statistically significant, there was a clear trend towards higher miRNA abundance in milk‐derived EVs (*p* < 0.10).

When samples were stratified by farm (L, S and Z), the highest miRNA abundance was observed in milk‐EVs from Farm Z (13.9 ± 7.5 million), followed by Farm L (7.9 ± 2.4 million) and Farm S (6.5 ± 2.4 million).

**FIGURE 4:**
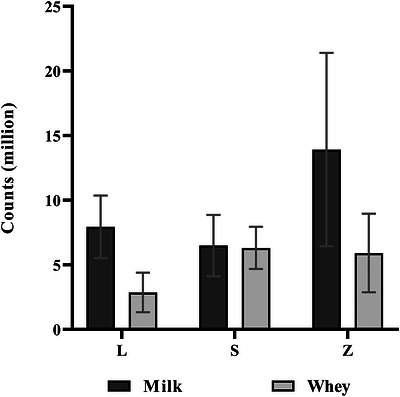
Filtered miRNA mean read counts (in millions ± SD) in milk and whey‐EVs across the three farms (L, S and Z).

In contrast, whey‐EVs showed lower or comparable values across farms, ranging from 2.9 ± 1.5 million (Farm L) to 6.3 ± 1.6 million (Farm S) (Figure 4). No significant differences in miRNA abundance were detected among farms within the same matrix.

However, paired matrix comparisons within farms revealed a significant difference in Farm L, where milk‐EVs contained significantly more miRNAs than whey‐EVs (*p* < 0.05). Overall, these results indicate that milk consistently carries a higher miRNA load than whey, with moderate but noticeable variation among farms.

### 3.5 Identification of the Most Abundant miRNAs

The 10 most abundant miRNAs were identified based on their average CPM values in milk and whey‐EVs. The top 10 miRNAs detected in both matrices representing the upper 25th percentile of the dataset were bta‐let‐7a‐5p, bta‐let‐7b, bta‐miR‐148a, bta‐miR‐200c, bta‐miR‐26a, bta‐miR‐26c, bta‐miR‐30d, bta‐miR‐3596 and bta‐miR‐99a‐5p. Among these, bta‐miR‐151‐3p was more abundant in milk‐EVs, whereas bta‐miR‐21‐5p showed higher expression levels in whey‐EVs (Table 2). Notably, bta‐miR‐151‐3p was among the most abundant miRNAs in milk but was not detected in whey samples, while bta‐miR‐21‐5p showed the opposite pattern. These differences reflect matrix‐specific abundance variation rather than differential expression analysis, as Table 2 reports mean CPM values without applying statistical thresholds.

**TABLE 2:** Top 10 most abundant miRNAs identified in milk and whey‐EVs based on mean counts per million (CPM).

miRNA	CPM_Milk	CPM_Whey
bta‐miR‐148a	566676.04	476709.21
bta‐miR‐99a‐5p	72544.30	46412.60
bta‐let‐7b	34469.98	45736.21
bta‐miR‐3596	34469.72	45735.77
bta‐let‐7a‐5p	33636.09	52942.96
bta‐miR‐26a	25509.15	27589.74
bta‐miR‐26c	25508.06	27589.63
bta‐miR‐30d	24518.15	24099.46
bta‐miR‐200c	23703.93	30468.81
bta‐miR‐151‐3p	22525.13	nd
bta‐miR‐21‐5p	nd	26790.58

*Note*: Values represent average expression levels across biological replicates. Data are presented as mean CPM values calculated from normalised read counts (*n* = 9 per matrix).

Abbreviation: nd, not detected (no measurable CPM across biological replicates after normalisation).

To further characterise the exosomal miRNA composition, the 10 most abundant miRNAs were identified separately for milk and whey‐EVs across the three farms. Milk‐EVs exhibited a largely conserved high‐abundance miRNA core, consistently dominated by bta‐miR‐148a, bta‐miR‐99a‐5p and several members of the let‐7 and miR‐26 families. In contrast, whey‐EVs displayed a slightly more heterogeneous high‐abundance profile, with bta‐miR‐21‐5p appearing among the top‐ranked miRNAs only in this matrix. Bta‐miR‐148a was the most abundant miRNA in all three farms, consistently enriched in both milk and whey‐EVs, particularly in Farm Z, which had the highest overall miRNA content. Other miRNAs frequently among the top‐expressed profiles included bta‐miR‐99a‐5p, bta‐let‐7a‐5p, bta‐let‐7b, bta‐miR‐26a, bta‐miR‐26c, bta‐miR‐200c, bta‐miR‐30d and bta‐miR‐3596.

### 3.6 Comparative Stability Analysis Among Collections (Stability *Z*‐Score)

**FIGURE 5:**
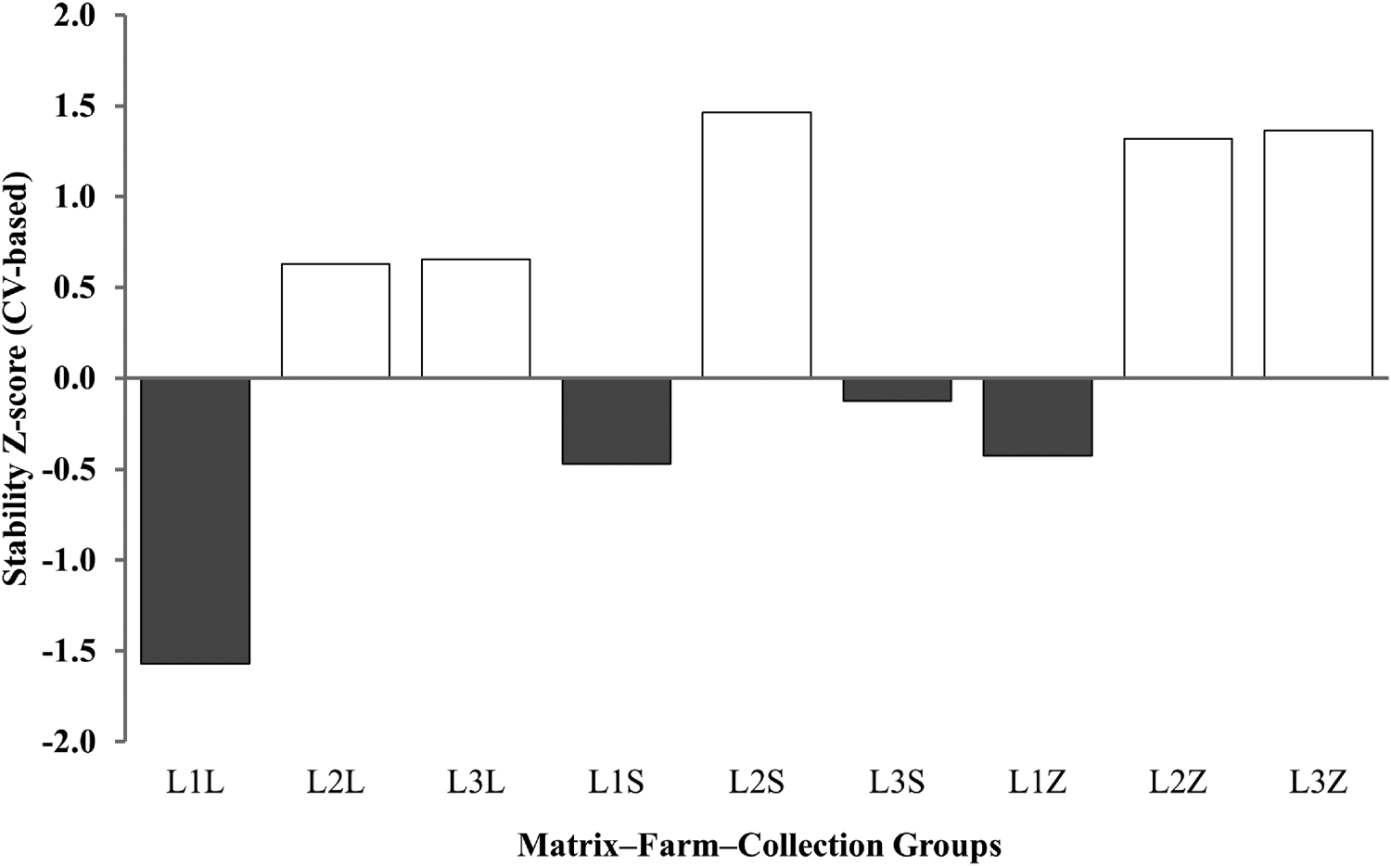
Stability *Z*‐scores of EV miRNA profiles in milk samples across farms (L, S, Z) and collections (1–3). *Z*‐scores were calculated from the normalised coefficient of variation of mean CPM values for priority miRNAs. Negative *Z*‐scores (grey bars) indicate greater sample stability and reproducibility, while positive *Z*‐scores (white bars) indicate increased variability.

**FIGURE 6:**
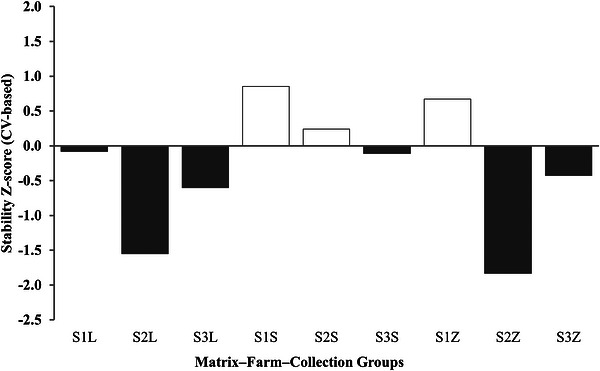
Stability *Z*‐scores of EV miRNA profiles in whey samples across farms and collections. *Z*‐scores were derived from the normalised coefficient of variation of mean CPM values for priority miRNAs. More negative *Z*‐scores indicate greater stability and reproducibility of miRNA expression within whey‐EV samples. Grey bars indicate the most stable collections, reflecting optimal RNA quality.

A comparative stability analysis across farms and collections revealed significant differences in the consistency ofEV miRNA expression. Stability *Z*‐scores highlighted collection‐dependent variability in both milk and whey‐EVs, with some farm‐collection groups exhibiting low (negative) *Z*‐scores, indicating highly reproducible miRNA profiles, and others showing positive *Z*‐scores, reflecting greater variability. Figures 5 and 6 summarise the Stability *Z*‐score distributions for milk and whey, respectively.

Milk‐ EV miRNAs exhibited substantial variability across farm–collection groups. Several combinations, particularly L2S, L2Z and L3Z, had strongly positive *Z*‐scores, indicating reduced reproducibility of their high‐abundance miRNA profiles. Conversely, groups such as L1L, L1S and L1Z had markedly negative *Z*‐scores, reflecting high stability (Figure 5).

Whey‐EV miRNAs exhibited a more balanced stability distribution. Groups S2L, S3L, S2Z and S3Z had strongly negative *Z*‐scores, indicating highly consistent expression profiles, while S1S and S1Z had positive *Z*‐scores and increased variability (Figure 6). Overall, whey‐EVs displayed a mixed but more evenly distributed stability pattern compared with milk.

These data confirm that stability varies substantially among farm–collection combinations within each matrix and is not consistent across sampling conditions.

### 3.7 Differential Expression Analysis of EV miRNAs in Bovine Milk and Whey

**FIGURE 7:**
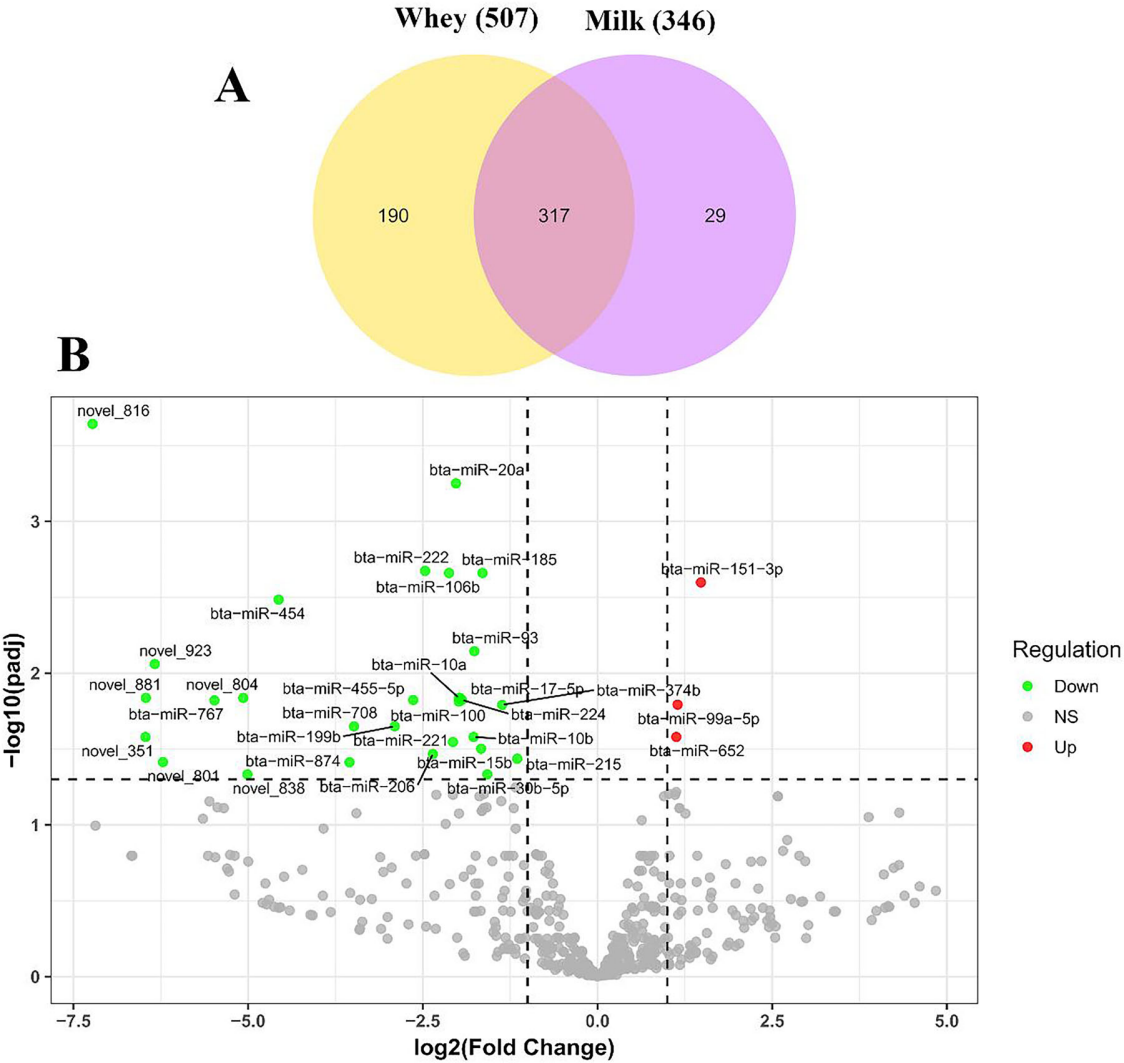
Comparative miRNA profiling between milk and whey‐EVs. (A) Venn diagram showing the distribution of miRNAs detected in milk‐ and whey‐derived EVs. A total of 317 miRNAs were shared between the two matrices, while 190 were uniquely detected in whey EVs and 29 were uniquely detected in milk EVs. (B) Volcano plot illustrating differentially expressed miRNAs between milk and whey EVs (milk *vs* whey). Red dots represent miRNAs significantly up‐regulated in milk, and green dots represent miRNAs significantly up‐regulated in whey (adjusted *p* < 0.05; |log_2_FC| ≥ 1).

Venn diagram analysis revealed substantial overlap in miRNA composition between milk and whey EVs, with 317 miRNAs shared between the two matrices, 190 detected exclusively in whey‐derived EVs, and 29 detected exclusively in milk‐derived EVs (Figure 7A).

DESeq2 identified 32 significantly modulated miRNAs between milk and whey‐EVs: 3 miRNAs were upregulated in milk, while 29 were more abundant in whey. The complete list of miRNAs is provided in Table S3; only significant (*p* < 0.05) miRNAs are shown in the volcano plot (Figure 7B).

**FIGURE 8:**
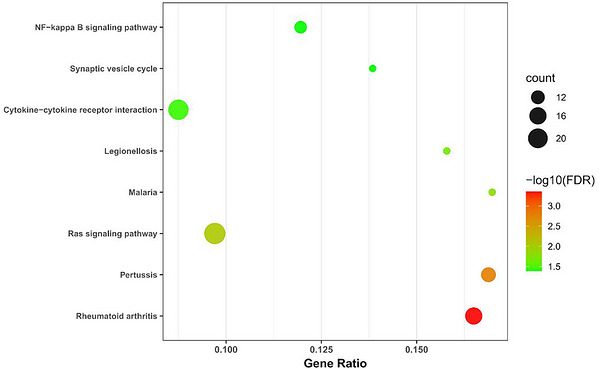
Bubble plot of KEGG pathway enrichment analysis for predicted target genes of differentially expressed miRNAs in the milk versus whey comparison. Bubble size indicates the number of enriched target genes (Hits), colour represents statistical significance (–log10(FDR)), and the x‐axis shows the GeneRatio (Hits/total genes in the input list). Only pathways passing the significance threshold are shown.

To investigate the potential biological roles of differentially expressed miRNAs, predicted target genes were subjected to KEGG pathway enrichment analysis. KEGG pathway enrichment analysis of the predicted targets of the 25 *Bos taurus* differentially expressed miRNAs between milk and whey‐EVs revealed significant over‐representation of several immune‐related and signalling pathways (FDR < 0.05). The most enriched pathways were Rheumatoid arthritis (16 hits, FDR = 0.00044) and Pertussis (13 hits, FDR = 0.0016), as well as broad regulatory cascades such as the Ras signalling pathway (23 hits, FDR = 0.0088) and NF‐kappa B signalling (11 hits, FDR = 0.0409). Additional enriched pathways included Cytokine‐cytokine receptor interaction, Malaria, Legionellosis and the Synaptic vesicle cycle. These results indicate that the differentially expressed miRNAs between milk and whey‐EVs predominantly target genes involved in immune regulation, inflammatory signalling and host–pathogen interaction (Figure 8). The genes contributing to the Synaptic vesicle cycle category included multiple components of vesicle trafficking and membrane fusion machinery, such as RAB3A, DNM1/2, clathrin adaptor complex subunits (AP2 family), clathrin chains (CLTC, CLTA/B), SNARE‐related proteins (STX1A, VAMP2) and several vacuolar ATPase subunits (ATP6V family). Complete KEGG enrichment results, including non‐significant pathways, are provided in Table S4.

### 3.8 Farm‐Dependent Differences in Milk‐EV miRNA

To assess whether farm‐specific factors influenced the composition of EV miRNAs independently of the biological matrix, differential expression analysis was conducted separately for milk and whey samples. The complete differential expression results for all comparisons are provided in Tables S5–S7. Significantly differentially expressed miRNAs are reported in Table 3.

**TABLE 3:** Farm‐dependent differentially expressed miRNAs in milk‐derived EVs with miRNA‐level GO Biological Process enrichment (STRING; FDR < 0.05).

Comparison	sRNA	log2FC	*p*adj	Representative enriched GO terms (STRING; FDR < 0.05)
L vs. S	bta‐miR‐204	−7.29	4.43E‐07	None
	bta‐miR‐205	−4.91	3.17E‐03	None
	bta‐miR‐34c	3.25	1.75E‐03	None
	bta‐miR‐370	−7.05	3.17E‐03	Vesicle‐mediated transport (GO:0016192)
	bta‐miR‐450b	−3.85	4.06E‐02	None
	bta‐miR‐654	−6.71	4.37E‐02	None
	bta‐miR‐9‐3p	−10.29	2.38E‐02	None
	bta‐miR‐9‐5p	−4.43	1.86E‐02	None
	novel_50	−2.90	3.29E‐03	None
Z vs. S	bta‐miR‐124a	8.33	5.43E‐04	None
	bta‐miR‐205	−3.59	1.66E‐02	None
	bta‐miR‐2904	−2.36	1.53E‐02	None
	bta‐miR‐323	−4.73	7.44E‐03	None
	novel_261	−3.97	3.65E‐02	None
	novel_50	−4.38	4.76E‐09	None

*Note*: ‘None’ indicates no significant enrichment after correction.

#### 3.8.1 Farm L Versus Farm S

Within milk‐derived EVs, the comparison between Farms L and S revealed a limited but biologically meaningful set of differentially expressed miRNAs. Differential expression analysis identified nine miRNAs significantly modulated between the two farms (adjusted *p* value < 0.05 and |log_2_FC| ≥ 1). Only bta‐miR‐34c was upregulated in Farm L, whereas eight miRNAs (bta‐miR‐204, bta‐miR‐205, bta‐miR‐370, bta‐miR‐450b, bta‐miR‐654, bta‐miR‐9‐3p, bta‐miR‐9‐5p and novel_50) were more abundant in Farm S (Table 3). At the individual miRNA level, GO Biological Process enrichment analysis (STRING; FDR < 0.05) identified significant enrichment only for bta‐miR‐370, which was associated with vesicle‐mediated transport, while no significant GO terms were detected for the remaining miRNAs (Table 3). Several of these miRNAs, including miR‐9‐3p and miR‐204, have previously been implicated in epithelial signalling programmes and epithelial‐to‐mesenchymal transition processes (Ding et al. 2017). In addition, miR‐204 (Kassan et al. 2017) and miR‐370 (Li et al. 2021) have been reported to participate in cellular stress adaptation and immune signalling pathways.

To assess the functional implications of these differences, KEGG pathway enrichment analysis was performed on the predicted targets of the differentially expressed miRNAs. Due to the limited number of *Bos taurus* miRNAs with annotated targets (*n* = 8) and low target‐gene convergence, no KEGG pathways reached significance after FDR correction. This indicates that farm‐dependent differences in individual miRNAs did not result in coherent pathway‐level enrichment, consistent with the small and functionally heterogeneous set of DE miRNAs.

#### 3.8.2 Farm Z Versus Farm L

The comparison between Farms Z and L identified no significantly differentially expressed miRNAs that met the predefined thresholds (adjusted *p* < 0.05 and |log_2_FC| ≥ 1). Although moderate expression shifts were observed for several miRNAs, none reached statistical significance (Table S6). Therefore, KEGG enrichment analysis was not conducted for this contrast.

#### 3.8.3 Farm Z Versus Farm S

The comparison between Farms Z and S identified six significantly modulated miRNAs (adjusted *p* < 0.05 and |log_2_FC| ≥ 1) (Table 3). bta‐miR‐124a was strongly upregulated in Farm Z, while bta‐miR‐205, bta‐miR‐2904, bta‐miR‐323 and two novel miRNAs (novel_261 and novel_50) were more abundant in Farm S. This pattern reveals clear farm‐dependent divergence in specific regulatory miRNAs, with bta‐miR‐124a showing the greatest positive fold change, and novel_50 and bta‐miR‐323 showing the largest increases in Farm S.

The complete list of DE miRNAs is provided in Table S7.

KEGG enrichment analysis was conducted on the four *Bos taurus* miRNAs with annotated targets; however, no pathway reached statistical significance after FDR correction, reflecting the small size of the miRNA set and low overlap among their predicted targets.

### 3.9 Farm‐Dependent Differences in Whey EV miRNAs

The complete differential expression results of EV miRNAs in whey samples for all comparisons are provided in Tables S8–S10. Significantly differentially expressed miRNAs are listed in Table 4.

**TABLE 4:** Farm‐dependent differentially expressed miRNAs in whey‐derived EVs with miRNA‐level GO Biological Process enrichment (STRING; FDR < 0.05).

Comparison	sRNA	log2FC	*p*adj	Representative enriched GO terms (STRING; FDR < 0.05)
L vs. S	bta‐miR‐11988	19.89	5.10E‐04	None
	bta‐miR‐130a	20.37	1.44E‐04	None
	bta‐miR‐154b	19.83	2.02E‐04	None
	bta‐miR‐17‐5p	5.56	2.50E‐04	Immune response (GO:0006955); Regulation of leukocyte activation (GO:0002694); Cytokine‐mediated signaling pathway (GO:0019221); Response to lipopolysaccharide (GO:0032496)
	bta‐miR‐21‐3p	19.75	5.10E‐04	None
	bta‐miR‐31	9.25	1.86E‐02	None
	bta‐miR‐34a	9.76	1.12E‐02	None
	bta‐miR‐494	8.84	4.19E‐02	None
	novel_643	19.83	2.02E‐04	None
	novel_776	21.54	1.56E‐06	None
	novel_935	20.42	1.44E‐04	None
Z vs. L	bta‐miR‐10a	−2.86	1.56E‐02	None
	bta‐miR‐17‐5p	−2.33	4.52E‐02	Regulation of leukocyte activation (GO:0002694); Immune response (GO:0006955); Regulation of cell activation (GO: 0050865)
	bta‐miR‐2284x	3.07	1.56E‐02	None
	bta‐miR‐455‐5p	−3.82	1.56E‐02	None
	bta‐miR‐487b	−8.49	2.29E‐02	None
	bta‐miR‐494	−3.84	1.56E‐02	None
	bta‐miR‐654	−5.16	3.61E‐02	None
	bta‐miR‐9‐3p	−3.51	1.56E‐02	None
	bta‐miR‐9‐5p	−3.40	1.56E‐02	None
	bta‐miR‐93	−2.28	2.06E‐02	None
	bta‐miR‐95	−3.01	1.56E‐02	None
	novel_351	−7.36	1.56E‐02	None
Z vs. S	bta‐miR‐302a	−8.99	4.75E‐02	None
	bta‐miR‐302d	−8.99	4.75E‐02	None

*Note*: ‘None’ indicates no significant enrichment after correction.

#### 3.9.1 Farm L Versus Farm S

The comparison of whey‐derived EVs from Farms L and S revealed a substantial number of differentially expressed miRNAs. Using DESeq2 with an adjusted *p* < 0.05 and |log_2_FC| ≥ 1, 11 miRNAs were identified as significantly modulated, all showing markedly higher expression in samples from Farm L. These included eight *Bos taurus* miRNAs (bta‐miR‐11988, bta‐miR‐130a, bta‐miR‐154b, bta‐miR‐17‐5p, bta‐miR‐21‐3p, bta‐miR‐31, bta‐miR‐34a and bta‐miR‐494) and three novel miRNAs (novel_643, novel_776 and novel_935) (Table 4). At the individual miRNA level, GO Biological Process enrichment analysis (STRING; FDR < 0.05) identified significant enrichment only for bta‐miR‐17‐5p, which was associated with immune‐related processes including immune response, regulation of leukocyte activation, cytokine‐mediated signalling pathway and response to lipopolysaccharide (Table 4). No significant GO terms were detected for the remaining miRNAs after correction. Notably, several miRNAs exhibited extremely high fold changes (log_2_FC > 19), reflecting profound differences in the EV miRNA cargo between the two farms. The complete list of DE miRNAs is provided in Table S8.

To investigate the functional implications of the miRNA differences observed between Farms L and S in whey‐derived EVs, KEGG pathway enrichment analysis was performed on the predicted targets of the eight *Bos taurus* miRNAs identified as differentially expressed.

In contrast to the milk‐derived comparison, this analysis revealed several significantly enriched pathways (FDR < 0.05), indicating coordinated modulation of specific regulatory modules.

**FIGURE 9:**
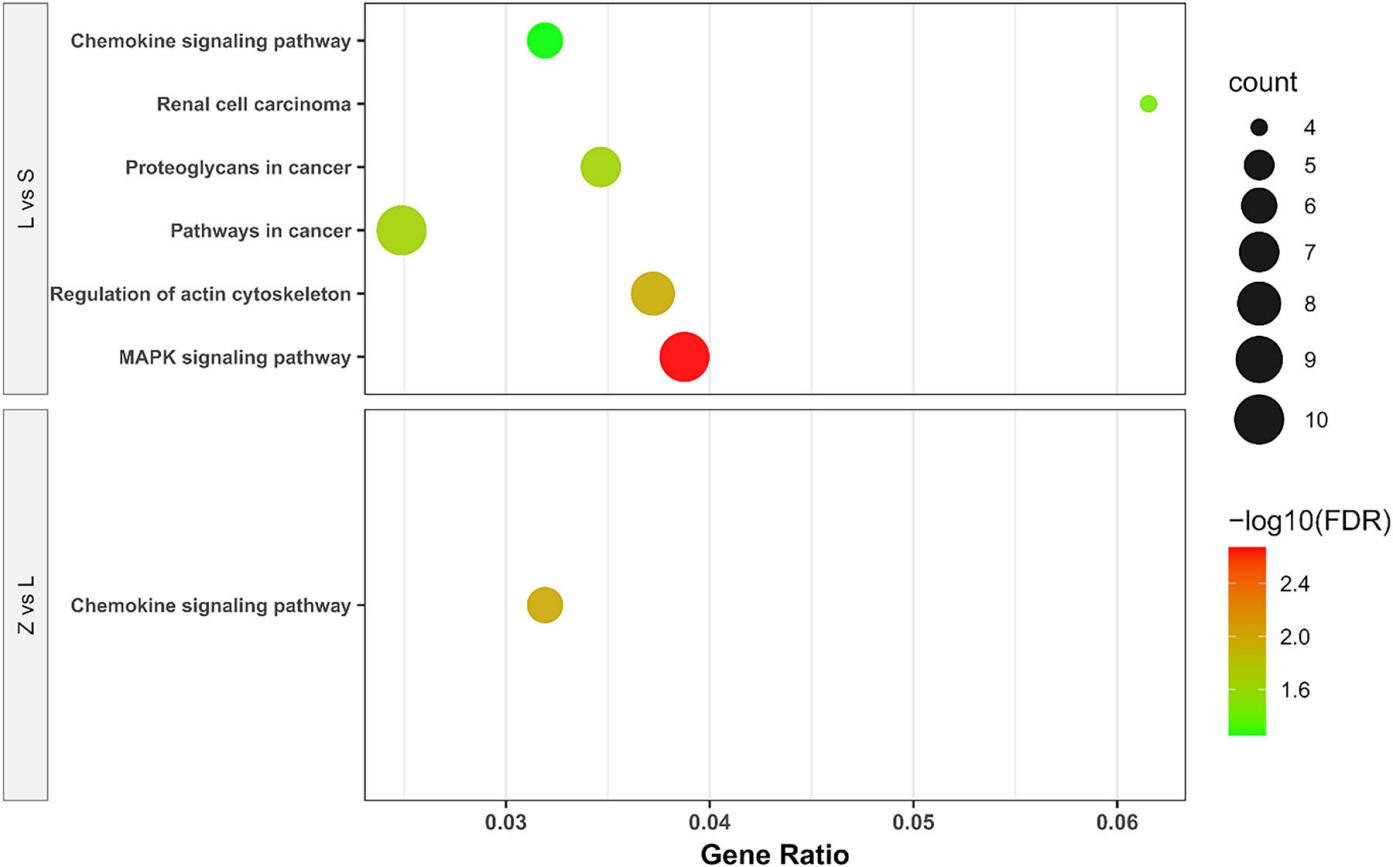
Bubble plot of KEGG pathway enrichment analysis for predicted target genes of significantly differentially expressed miRNAs in milk‐EVs. Results are shown for the L versus S and Z versus S farm comparisons. Each bubble represents a KEGG pathway; bubble size indicates the number of enriched target genes (count) and colour reflects statistical significance (–log10(FDR)). The x‐axis shows the GeneRatio (number of enriched genes relative to the total number of input genes). Only pathways reaching statistical significance are displayed. KEGG pathway enrichment summary for differentially expressed miRNAs between farms L and S (whey‐EVs).

The most significantly enriched pathway was the MAPK signalling pathway (10 hits, FDR = 0.0021), a central regulator of cellular proliferation, stress responses and immune signalling. Other enriched pathways included regulation of actin cytoskeleton (FDR = 0.0109), pathways in cancer, proteoglycans in cancer (FDR = 0.0221 for both) and renal cell carcinoma (FDR = 0.0346). The top entries are shown in Figure 9.

#### 3.9.2 Farm Z Versus Farm L

The comparison between Farms Z and L in whey‐derived EVs revealed a clear asymmetry in miRNA abundance. Using DESeq2 with adjusted *p* < 0.05 and |log_2_FC| ≥ 1, 11 miRNAs were identified as significantly differentially expressed, all showing higher expression in Farm L (Table 4). These included 10 *Bos taurus* miRNAs (bta‐miR‐10a, bta‐miR‐17‐5p, bta‐miR‐2284x, bta‐miR‐455‐5p, bta‐miR‐487b, bta‐miR‐494, bta‐miR‐654, bta‐miR‐9‐3p, bta‐miR‐9‐5p, bta‐miR‐93, bta‐miR‐95) and one novel miRNA (novel_351).

Several of these miRNAs, particularly bta‐miR‐9‐3p, miR‐9‐5p, miR‐654 and miR‐455‐5p, displayed large negative log_2_ fold changes, indicating markedly higher levels in Farm L compared with Farm Z. Notably, bta‐miR‐17‐5p again showed significant GO Biological Process enrichment (STRING; FDR < 0.05) for immune‐related pathways, consistent with the pattern observed in the L versus S comparison (Table 4).

The complete list of DE miRNAs is provided in Table S9.

To assess whether the miRNA differences observed between Farms Z and L in whey‐EVs were associated with coordinated functional signatures, KEGG pathway enrichment analysis was performed on the predicted targets of the 10 *Bos taurus* miRNAs identified as differentially expressed. Unlike the milk‐derived comparisons, this analysis identified only one significantly enriched pathway after FDR correction: the Chemokine signalling pathway (FDR < 0.05) (Figure 9; Table S10). This pathway plays a central role in regulating leukocyte activation, migration and inflammatory signalling, suggesting that the miRNAs upregulated in Farm L may influence immune‐related communication within whey‐EVs.

The enrichment of chemokine‐related targets aligns with the regulatory functions attributed to miRNAs such as miR‐9‐3p, miR‐17‐5p, miR‐93 and miR‐654, which are known to modulate immune and stress‐response pathways (Figure 9; Table S11). These findings indicate that, although differences between Farm Z and Farm L were limited to a small set of miRNAs, their predicted targets converge on a biologically coherent signalling module.

#### 3.9.3 Farm Z Versus Farm S

The comparison of whey‐EVs from Farms Z and S revealed a very limited set of differentially expressed miRNAs (Table S12). Using DESeq2 with adjusted *p* < 0.05 and |log_2_FC| ≥ 1, two miRNAs (bta‐miR‐302a and bta‐miR‐302d) were found to be significantly more abundant in Farm S, with no miRNAs upregulated in samples from Farm Z (Table 4). Both miR‐302a and miR‐302d exhibited extremely large negative fold changes (log_2_FC ≈ –9), indicating their near absence in Farm Z and high abundance in Farm S. These miRNAs belong to miR‐302 family, a cluster with established regulatory roles in cell proliferation, reprogramming and developmental signalling.

Although both miR‐302a and miR‐302d are well‐characterised members of the miR‐302/367 cluster in several mammalian species, these miRNAs are not annotated in *Bos taurus* in the miRBase or miRNet reference databases. Consequently, no bovine target genes are available for pathway analysis, and KEGG enrichment could not be performed for this contrast. This limitation reflects current gaps in bovine miRNA annotation rather than a lack of biological relevance.

## 4 Discussion

The EV isolation workflow produced high‐quality small EV preparations that met MISEV2023 criteria (Welsh et al. 2024). Particle size distribution, morphology and marker expression were consistent with previous descriptions of bovine (Colitti et al. 2019; Melnik et al. 2021) and human milk EVs (van Herwijnen et al. 2018), confirming the robustness of the isolation procedure and the consistency of the recovered EV fraction. The high particle‐to‐protein ratio (∼10^10^ particles/µg protein) further supports the purity of the samples and indicates their suitability for downstream molecular analyses, including miRNA sequencing, an important prerequisite for accurate EV‐miRNA profiling (Zempleni et al. 2017).

The significantly higher protein concentration observed in whey‐derived EV preparations likely reflects intrinsic differences in matrix composition. Whey contains abundant soluble proteins, including lactoferrin, β‐lactoglobulin and α‐lactalbumin, which may partially co‐isolate during ultracentrifugation despite clarification steps (Sedykh et al. 2019; Kleinjan et al. 2021). Although the particle‐to‐protein ratio remained comparable between milk and whey EVs, the higher absolute protein content in whey preparations may indicate either increased vesicle‐associated protein cargo or partial co‐isolation of soluble whey proteins.

These differences have implications for EV purity assessment and cargo interpretation. While whey offers practical advantages for large‐scale EV recovery due to reduced lipid and casein interference, its protein‐rich composition may affect downstream proteomic analyses and functional interpretation. Therefore, comparisons between milk‐ and whey‐derived EVs should consider matrix‐specific protein background as a potential contributing factor.

From an application‐oriented perspective, both milk‐ and whey‐derived EVs offer distinct advantages. Whole milk is a complex biological matrix that preserves interactions between vesicles and lipid or casein components, which may be relevant in physiological and nutritional contexts. In contrast, whey, a by‐product of cheese‐making, provides a more manageable and scalable matrix with reduced lipid interference, supporting efficient EV recovery for industrial or translational applications. Our findings are consistent with recent work by del Saz‐Lara et al. (2025), who demonstrated the feasibility of large‐scale EV isolation from milk and cheesemaking whey, highlighting the translational relevance of comparative profiling between these matrices. Importantly, the direct comparison between milk‐ and whey‐derived EVs is a central aspect of this study. Although whey is produced from milk during cheese‐making, the two matrices differ substantially in lipid content, casein micelles, and soluble protein composition. Evaluating whether EV yield, miRNA cargo composition, and functional signatures are preserved or altered during matrix separation is essential both biologically and practically. This comparison distinguishes intrinsic vesicle‐associated signals from matrix‐dependent effects and informs the suitability of whey as a scalable and sustainable alternative source of EVs for biomarker discovery and downstream applications.

Between farms and matrices, we identified a conserved set of highly abundant miRNAs: bta‐miR‐148a, miR‐26a, miR‐200c and several members of the let‐7 family, consistent with previous reports in ruminants and humans (Chen et al. 2010; Sun et al. 2016; Leiferman 2018; Shang et al. 2023). Many of these miRNAs are central regulators of epithelial homeostasis, immune modulation and developmental signalling, reinforcing the emerging concept that milk‐derived EVs serve as carriers of bioactive regulatory RNAs with potential physiological relevance for both the mammary gland and the neonate (Sedykh et al. 2019; Reif et al. 2020). Collectively, these findings support the biological relevance of conserved miRNA cargo in bovine milk‐derived EVs.

A key finding is that variability in EV miRNA expression was influenced more by farm‐ and collection‐specific factors than by the biological matrix (milk *vs* whey). Stability *Z*‐score analysis revealed heterogeneous patterns in milk samples, ranging from highly reproducible to highly variable profiles, while whey samples showed more uniform stability with several highly consistent groups. These differences likely reflect underlying farm‐level variables such as management practices, nutrition, environmental stressors or microbial load (Reinhardt et al. 2012; Sun et al. 2015; Colitti et al. 2019). Pre‐analytical variables specific to milk, including the time from milking to freezing, temperature fluctuations during collection, efficiency of fat removal, and casein precipitation steps, may further influence EV recovery and RNA integrity. Industrial processing factors such as homogenisation and heat exposure may also affect vesicle stability and cargo composition. Notably, the identification of highly stable farm–collection combinations demonstrates that EV miRNA profiling can be reproducible when pre‐analytical sources of variation are controlled.

Matrix‐specific differences were further supported by differential expression analysis between milk and whey EVs, which identified 32 significantly modulated miRNAs. KEGG enrichment revealed a strong over‐representation of immune‐related pathways, including NF‐κB signalling, cytokine‐receptor interactions, chemokine signalling and Ras signalling pathways, which are core hubs in epithelial‐immune communication (Mutai et al. 2020; Verma et al. 2022). KEGG categories associated with infectious diseases (Pertussis, Malaria, Legionellosis) appeared as expected from the clustering of innate immunity genes within these modules and do not indicate pathogen‐specific activity. Although annotated as ‘Synaptic vesicle cycle’, the enriched genes in this category predominantly encode conserved components of vesicle trafficking and membrane fusion machinery, including Rab GTPases, clathrin adaptor complex subunits, dynamins, SNARE‐associated proteins and vacuolar ATPase subunits involved in endosomal acidification. These proteins are not neuron‐specific but represent core elements of vesicle budding and fusion pathways, which are also implicated in EV biogenesis and secretion, suggesting potential differences in vesicle trafficking dynamics between matrices. These observations support the interpretation that differences in vesicle trafficking and membrane dynamics may contribute to matrix‐dependent variation in EV cargo composition, with potential downstream implications for epithelial and immune‐related signalling (Melnik et al. 2021; Yang et al. 2024; Wang et al. 2025).

Farm‐specific comparisons revealed small but biologically coherent signatures. In milk‐EVs, Farm S showed enrichment of miRNAs such as miR‐204, miR‐205, miR‐9‐3p and miR‐370, which modulate epithelial integrity, immune signalling and oxidative stress response (Ma et al. 2011; Chen et al. 2017). Farm *Z* samples displayed high levels of miR‐124a, a regulator of inflammatory and differentiation pathways in epithelial tissues (Sun et al. 2016).

Farm‐level differences were even more pronounced in whey‐EVs, with Farm L showing strong upregulation of immune‐modulatory miRNAs, including miR‐17‐5p, miR‐31, miR‐34a and miR‐494. KEGG enrichment revealed significant activation of the chemokine signalling pathway, indicating convergence on inflammatory and cell migration regulatory modules (Sokol and Luster 2015). These results suggest that the miRNAs upregulated in Farm L whey‐EVs converge on molecular pathways involved in cell signalling, cytoskeletal dynamics and proliferation. In contrast, the Z versus S comparison identified only two differentially expressed miRNAs (miR‐302a and miR‐302d), which were strongly enriched in Farm S. Although these miRNAs are central to the pluripotency‐associated miR‐302/367 cluster (Greer Card et al. 2008), their lack of bovine annotation prevented reliable functional inference.

Several limitations must be acknowledged. Bovine miRNA annotation remains incomplete, leaving many novel or cross‐species miRNAs without validated targets, which restricts pathway analysis. Triplicate sampling enabled robust comparisons, but larger cohorts would improve statistical resolution, and analysis of individual cows would allow exploration of animal‐level variables such as lactation stage, parity and diet.

An additional consideration concerns pre‐analytical handling and storage conditions. Whole milk samples were stored at −20°C before complete removal of fat and casein components. Freeze–thaw cycles may disrupt fat globule membranes and associated lipid–protein complexes, potentially affecting the composition of the recovered EV fraction. In contrast, whey samples with lower lipid content may be less affected by freeze–thaw–induced structural alterations. Although all samples were stored under identical conditions, matrix‐specific differences in fat content represent a potential confounding factor in milk–whey comparisons, particularly when interpreting matrix‐dependent differences.

In addition, total RNA was extracted directly from EV preparations without prior RNase digestion of intact vesicles. Although this approach followed sequencing provider recommendations and was applied consistently across all samples, it does not distinguish between vesicle‐encapsulated and externally associated miRNAs. Future studies incorporating RNase protection assays could further refine the distinction between protected and surface‐associated RNA species. Finally, functional enrichment relied on predicted targets (miRanda); although comprehensive, these predictions would benefit from integration with mRNA‐seq or proteomic data to validate miRNA–mRNA interactions, thereby strengthening mechanistic interpretation.

## 5 Conclusion

In conclusion, this study provides an integrated, high‐resolution characterisation of EV‐associated miRNAs from bovine milk and whey. Both matrix‐ and farm‐specific factors influence the EV RNA cargo, with conserved immunoregulatory miRNAs predominating. The identification of stable farm–collection combinations and matrix‐dependent functional patterns offers a framework for improving sampling standardisation and highlights the potential regulatory roles of milk‐derived EVs in epithelial and immune processes. These findings enhance our understanding of bovine small extracellular vesicle biology and support future applications in biomarker discovery, animal health and nutritional research, particularly in the context of scalable EV recovery from dairy by‐products.

## Author Contributions

**Caterina Trevisan**: methodology, data curation, investigation, formal analysis. **Giulia Polacchini**: methodology, validation. **Bruno Stefanon**: data curation, revised paper. **Monica Colitti**: project administration, formal analysis, writing – original draft.

## Funding

This work was supported by the A1.3.1 PR FESR 2021 – 2027 Friuli‐Venezia Giulia Region under grant number 2024/2950.

## Conflicts of Interest

The authors report no conflicts of interest.

## Supporting information



Supporting Information: jex270165‐sup‐0001‐TableS1.xlsx

Supporting Information: jex270165‐sup‐0002‐TableS2.xlsx

Supporting Information: jex270165‐sup‐0003‐TableS3.xlsx

Supporting Information: jex270165‐sup‐0004‐TableS4.xlsx

Supporting Information: jex270165‐sup‐0005‐TableS5.xlsx

Supporting Information: jex270165‐sup‐0006‐TableS6.xlsx

Supporting Information: jex270165‐sup‐0007‐TableS7.xlsx

Supporting Information: jex270165‐sup‐0008‐TableS8.xlsx

Supporting Information: jex270165‐sup‐0009‐TableS9.xlsx

Supporting Information: jex270165‐sup‐0010‐TableS10.xlsx

Supporting Information: jex270165‐sup‐0011‐TableS11.xlsx

Supporting Information: jex270165‐sup‐0012‐TableS12.xlsx

## Data Availability

Raw sequencing data have been deposited in the NCBI Sequence Read Archive (BioProject ID: PRJNA1381537). The data that support the findings of this study are available from the corresponding author upon reasonable request.
